# Induction of Cell Death and Regulation of Autocrine Vitamin D Metabolism in Cervical Cancer by Physiological and GI20 Doses of 25-Hydroxycholecalciferol

**DOI:** 10.3390/ijms26094008

**Published:** 2025-04-24

**Authors:** Esther Zhou, Sachin Bhoora, Tahir S. Pillay, Rivak Punchoo

**Affiliations:** 1 Department of Chemical Pathology, University of Pretoria, Pretoria 0084, South Africa; evmzhou@gmail.com (E.Z.); tahir.pillay@up.ac.za (T.S.P.); 2 Division of Chemical Pathology, Department of Pathology, University of Cape Town, Cape Town 7935, South Africa; sachinbhoora808@gmail.com; 3 National Health Laboratory Service (NHLS), Tshwane Academic Division, Pretoria 0084, South Africa

**Keywords:** 25-hydroxycholecalciferol, vitamin D, vitamin D metabolizing system, cervical cancer, CYP27B1, CYP24A1, VDR, apoptosis

## Abstract

Vitamin D and its metabolites exert anti-cancer properties in various cancers; however, their effects on cervical cancer remain largely unexplored. To investigate this gap, we exposed HeLa adenocarcinoma cervical cells to physiological and the growth inhibition 20% (GI20) concentration of 25-hydroxycholecalciferol, the precursor hormone of active 1,25-dihydroxycholecalciferol. We then assessed its impact on cell health, and the expression of the genes and proteins involved in the activation and catabolism of vitamin D at the cellular level by autocrine vitamin D metabolism via the vitamin D metabolizing system (VDMS). Cell health was evaluated by crystal violet and alamarBlue assays, while cell cycle progression and apoptotic cell death markers were assessed by flow cytometry. Gross morphology and ultrastructure were observed using brightfield microscopy and transmission electron microscopy. Gene and protein analyses of the autocrine VDMS were assessed using reverse transcription polymerase chain reaction and Western blot, respectively. Our findings reveal that 25(OH)D_3_ inhibits cell growth and induces apoptosis in HeLa cervical cells in a dose-dependent manner through the autocrine upregulation of CYP27B1 and VDR. These autocrine effects most likely promote the bioactivation of 25(OH)D_3_ and intracellular signaling of pro-apoptotic genomic pathways by liganded VDR. Furthermore, the upregulation of CYP24A1 at GI20 treatment likely increases the catabolism of 25(OH)D_3_ and 1,25(OH)_2_D_3,_ and therefore may mitigate the anti-cancer action of the high-treatment dose. In summary, 25(OH)D_3_ holds immense potential as a complementary therapeutic treatment for cervical cancer.

## 1. Introduction

Vitamin D is widely known for stimulating calcium and phosphorus absorption, both of which are crucial for bone mineralization [1], but it also plays several other roles in the human body via endocrine and autocrine regulation by the vitamin D metabolizing system (VDMS). The autocrine VDMS consists of enzymes responsible for the activation and catabolism of vitamin D metabolites, and signaling via the vitamin D receptor (VDR). Vitamin D is activated in two steps by 25-hydroxylases (CYP2R1/CYP27A1) and by 25-hydroxyvitamin D-1α-hydroxylase (CYP27B1) to produce 25-hydroxycholecalciferol (25(OH)D_3_) and 1,25-dihydroxycholecalciferol (1,25(OH)_2_D_3_), respectively [2,3]. Both metabolites are catabolized by 24-hydroxylase (CYP24A1) and the products are excreted in bile [1,2]. The hormonally active 1,25(OH)_2_D_3_ binds to the vitamin D receptor (VDR) in target cells [3,4]. The VDR molecule then heterodimerizes with the retinoid X receptor (RXR) and the complex binds to vitamin D response elements (VDREs) in the promoter regions of target genes, thereby modulating gene expression [5,6]. Therefore, the autocrine regulation of the VDMS is important for maintaining cellular homeostasis and cell health [7].

The dsyregulation of the VDMS and vitamin D deficiency has been linked to various diseases, including cancer [1,8,9,10]. Vitamin D metabolites exert a negative effect on cancer cell health by inhibiting cell proliferation and inducing cell death through regulation by the VDMS in various cancers of the breast [11,12], prostate [13], lung [14], skin [15], colon [16,17], and cervix [18,19,20,21,22,23,24], demonstrated both in vitro and in vivo [2,25,26]. Vitamin D metabolites increase the expression of insulin-like growth factor-binding protein 3 (IGFBP-3) and cyclin-dependent kinase (CDK) inhibitors such as p21 and p27, thereby inhibiting cell proliferation through cell cycle arrest, in the G_1_ phase [27]. Hormonally active 1,25(OH)_2_D_3_ also inhibits telomerase activity and the Wnt/β-catenin signaling pathway, essential for adult tissue homeostasis [28]. In addition to cell health, the vitamin D metabolite induces apoptotic cell death by suppressing pro-survival proteins Bcl-2 and telomerase reverse transcriptase while simultaneously activating pro-apoptotic protein BAX [7]. Autophagic cell death has been demonstrated through the inhibition of mTOR 1,25(OH)_2_D_3_ in the MCF-7 and HL-60 cancer cell lines [6,29].

Although compelling evidence exists for using vitamin D in cancer treatment, the lack of clinical trials, randomized trial evidence, and inconsistent observational research remain a challenge in understanding the therapeutic potential of vitamin D in cancer treatment. In addition, insufficient evidence exists to elucidate the clinical effects of vitamin D on cervical cancer [30,31].

Cervical cancer is the fourth most prevalent cancer among women globally [32], with a disproportionate burden on women residing in low- and middle-income countries [33]. The histological type of cervical cancer, cervical adenocarcinoma, contributes to patient morbidity and mortality [34]. The burden is reinforced by socio-economic conditions, poor health care infrastructure, and comorbid illness [33].

As uterine cervical cancer tissue expresses the VDMS, investigating the regulation of the VDMS in cervical cancer is essential [35]. Friederich et al. have shown a functional VDMS in cervical cancer cells [18]. More recently, cholecalciferol and 25(OH)D_3_ have shown the regulation of the VDMS in cervical cancer cell lines [19,20,21,22,23]

The 25(OH)D_3_ metabolite is the gold standard biomarker in clinical medicine to evaluate vitamin D reserve in humans [36]. In addition to the systemic activation of 25(OH)D_3_ by the renal 1-alpha-hydroxylase enzyme, the autocrine VDMS activation of this pathway by 25(OH)D_3_ in cervical adenocarcinoma cancer cells is unexplored. The 25(OH)D_3_ metabolite is important to clinical dosing strategies and for monitoring vitamin D reserve in women with cervical cancer. Furthermore, the anti-cancer action role of 25(OH)D_3_ at the physiological level (with an upper limit of the human reference interval of 260 nM 25(OH)D_3_) [37] and supraphysiological dosing are unexplored in cervical adenocarcinoma.

As the anti-cancer effects of 25(OH)D_3_ on cervical adenocarcinoma remain unclear, this study aims to bridge this gap by investigating the impact of 25(OH)D_3_ treatments on cell growth, cell death, and VDMS gene and protein expression in the HeLa cervical adenocarcinoma cell line.

## 2. Results

### 2.1. Assessment of GI20 and Cell Health Parameters

**Figure 1:**
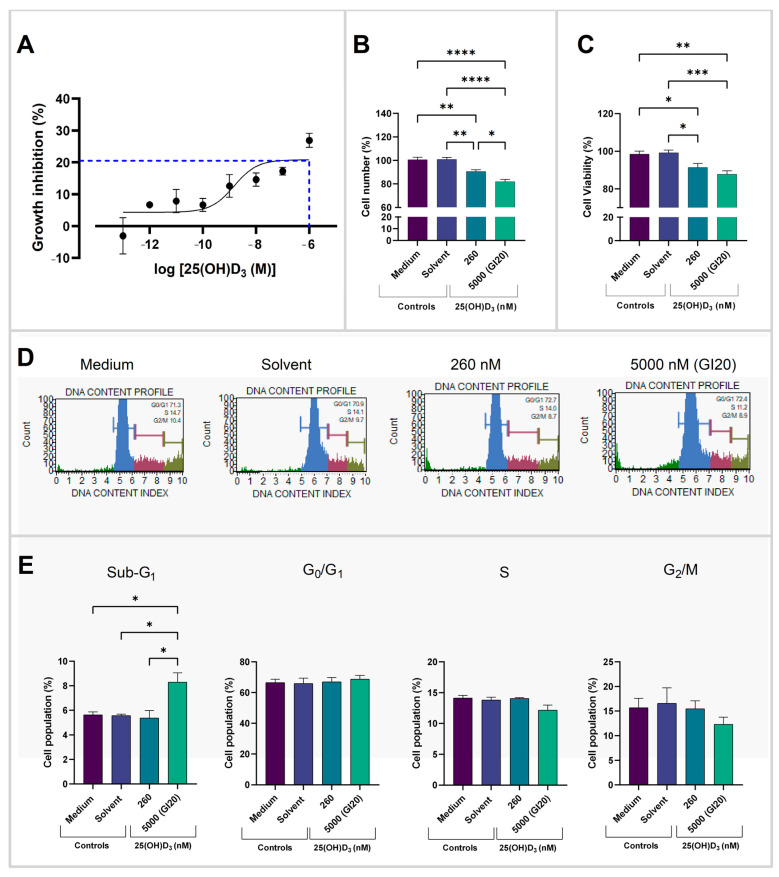
Growth inhibition curve and cell health parameters. (**A**) Growth inhibition curve of HeLa cells treated with log concentrations of 25(OH)D_3_. Growth inhibition at 5.0 × 10^−6^ M was 20% (blue dashed line). (**B**) Cell counts of HeLa cells enumerated with crystal violet staining and (**C**) cell viability quantified with the alamarBlue assay. (**D**) Histogram plots and (**E**) bar charts displaying percentage of HeLa cells in each phase of the cell cycle (sub-G_1_, G_0_/G_1_, S and G_2_/M). Data represent mean values from three biological replicates, each performed in technical triplicate. (* *p* < 0.05; ** *p* < 0.01; *** *p* < 0.001; **** *p* < 0.0001).

HeLa cells were treated with 25(OH)D_3_ with the aim of finding the growth inhibition concentration of 50%. Due to the toxicity of the solvent, 10-fold dilutions of 25(OH)D_3_ in the range of 5.0 × 10^−6^ M to 5.0 × 10^−13^ M were performed with the highest solvent dose concentration at 0.5% (*v*/*v*) ethanol. Growth inhibition of 22.90% ± 0.98% was observed at 5.0 × 10^−6^ M (5000 nM) 25(OH)D_3_. Extrapolation of the best-fit line showed 20% growth inhibition (GI20) at 5000 nM (Figure 1A). The cell health parameters showed a significant decrease in cell number (Figure 1B), which was observed at 5000 nM (*p* < 0.0001) and the 260 nM (*p* = 0.0016) 25(OH)D_3_ treatment doses. The two treatment doses also significantly differed in cell count (*p* = 0.0257). In addition, treatment with high dose (5000 nM) and low dose (260 nM) 25(OH)D_3_ significantly decreased cell viability relative to the solvent control (*p* = 0.0005 and *p* = 0.0156) (Figure 1C).

Cell cycle analysis (Figure 1D,E) demonstrated that treatment at 5000 nM significantly increased the sub-G_1_ cell population relative to the solvent (*p* = 0.0305), without any cell cycle arrest observed at G_0_/G_1_, S, G_2_/M cell populations.

### 2.2. 25(OH)D_3_ Induced Apoptotic Cell Death

**Figure 2:**
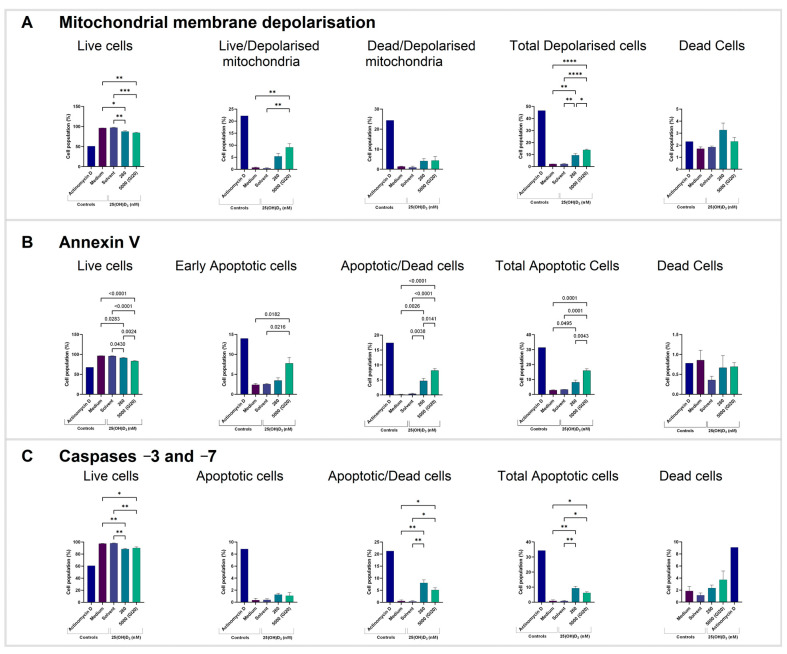
Detection of biochemical apoptosis with flow cytometric assays. Bar graphs of HeLa cell populations with (**A**) depolarized mitochondrial membrane potential, (**B**) externalized phosphatidylserine residues, (**C**) activated terminal caspases −3 and −7. Data represent mean values from three biological replicates. Dot plots are presented in Supplementary Figure S1. (* *p* < 0.05; ** *p* < 0.01; *** *p* < 0.001; **** *p* < 0.0001).

To evaluate early apoptosis, we employed flow cytometry to detect the percentage of cells with depolarized mitochondrial membrane potential. Physiological and GI20 dose treatments decreased the live cell population (*p* = 0.0050 and *p* = 0.0006, respectively). There was an increase in the live cell population with depolarized mitochondria (*p* = 0.0028) with the GI20 treatment dose (Figure 2A).

In addition, we assessed the externalization of phosphatidylserine residues on the outer leaflet of the cell membrane. Treatment at 5000 nM decreased the live cell population (*p* < 0.0001) and increased the early apoptotic, apoptotic and dead, and total apoptotic cell populations (*p* = 0.0216, *p* < 0.0001, and *p* = 0.0001, respectively). Likewise, the 260 nM treatment also decreased the live cell population (*p* < 0.0001) and increased the apoptotic and dead, and total apoptotic cell populations (*p* = 0.0038 and *p* = 0.0001, respectively) (Figure 2B).

**Figure 3:**
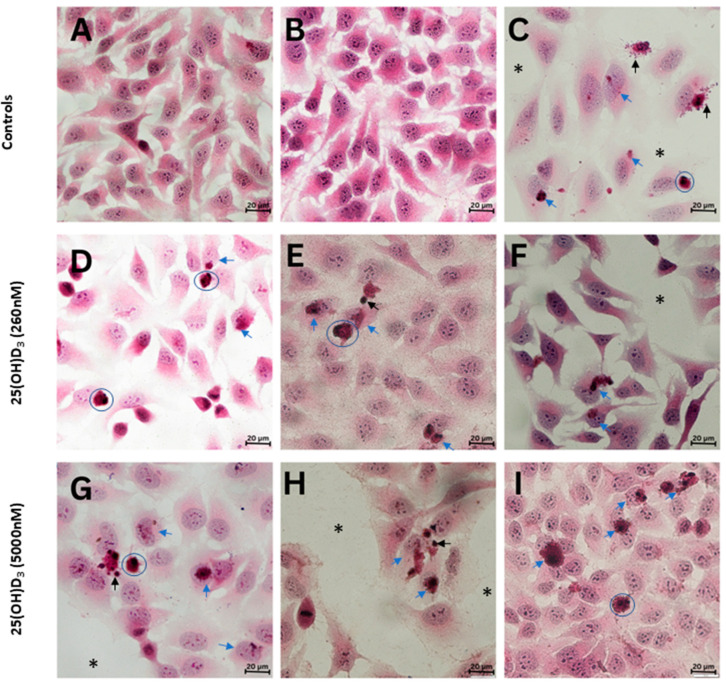
Brightfield microscopy images of HeLa cells stained with hematoxylin and eosin. Gross morphology of medium (**A**) and solvent (**B**) control showed clusters of healthy adenocarcinoma cells. The actinomycin D-treated positive control (**C**) showed decreased cell density (asterisks), cell blebbing and apoptotic bodies (black arrows), shrunken cytoplasm (blue circles), and nuclear and cytoplasm damage (blue arrows). Cultures treated with 25(OH)D_3_ at 260 nM (**D**–**F**) and 5000 nM (**G**–**I**) showed cells with hyper-condensed chromatin and nuclear damage (blue arrows), and apoptotic bodies and blebbing (black arrows). In addition, some experimental cultures showed round and shrunken cells (blue circles) and decreased cell density (asterisks). Scale bar = 20 µm.

Late apoptosis was analyzed by assessing the activity of executioner terminal caspases −3 and −7. Treatment with 5000 nM of 25(OH)D_3_ resulted in a decrease in the live cell population (*p* = 0.0095) and an increase in the apoptotic and dead cell population (*p* = 0.0345) (Figure 3C).

Brightfield microscopy was used to view the gross morphology of experimental and control HeLa cultures stained with hematoxylin and eosin. The notable features in 25(OH)D_3_-treated cells included cell membrane blebbing, apoptotic bodies, hyper-condensed chromatin (pyknosis), and cytoplasmic shrinkage (Figure 3C–F). In addition, experimental cultures had larger intercellular spaces in comparison to the control cultures.

**Figure 4:**
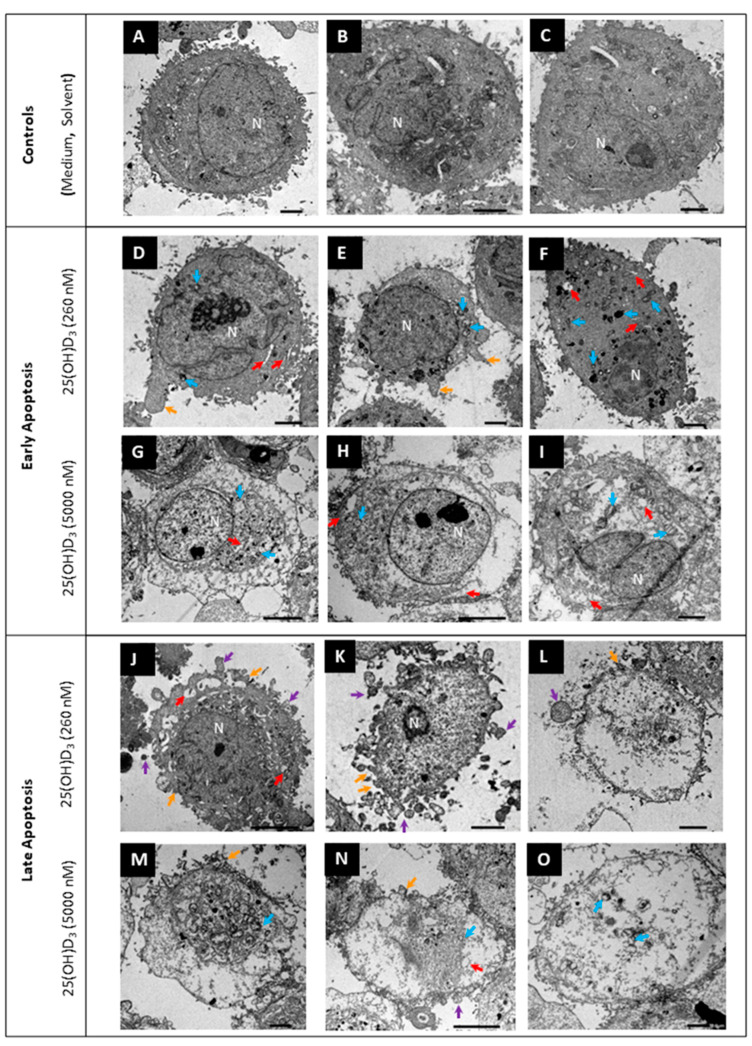
Ultrastructural analysis of HeLa cells by transmission electron microscopy. Control cultures (**A**–**C**), early apoptosis (**D**–**I**), and late apoptosis (**J**–**O**). Features of early apoptosis included abnormal mitochondria (light blue arrow) and intact cell membranes. Cells in the late stage of apoptosis showed shrunken (**I**,**J**) or indistinct nuclei (**L**–**O**), cell membrane blebbing (orange arrow), and apoptotic bodies (purple arrows). Some cells showed nuclear damage such as karyorrhexis (**D**) and pyknosis (**K**). In addition to features of apoptosis, dilated ER (red arrow) was present at both treatment doses. N, nucleus. Scale bar = 2.0 µm (**A**–**F**,**I**,**K**–**M**,**O**). Scale bar = 5.0 µm (**G**,**H**,**J**,**N**).

**Figure 5:**
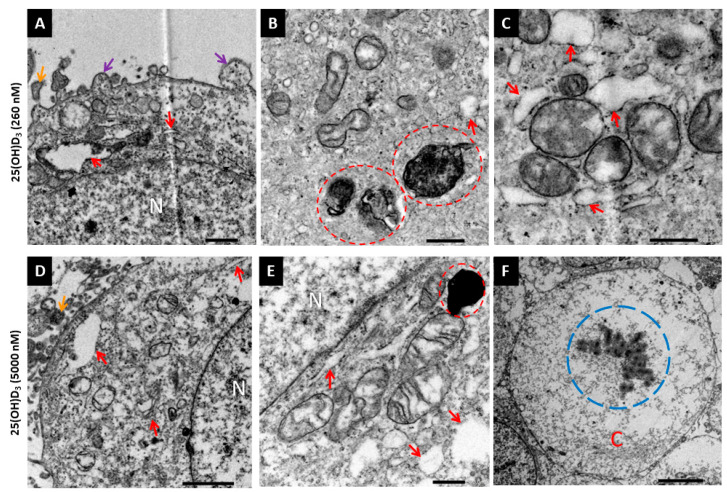
Ultrastructural analysis of 25(OH)D_3_-treated HeLa cells by transmission electron microscopy. HeLa cells treated with (**A**–**C**) 260 nM and (**D**,**E**) 5000 nM 25(OH)D_3_ showed features of apoptotic cell death. Mitochondria exhibited cristae disarrangements, partial or total breakdown of cristae (cristolysis), electron dense matrix (**C**), or electron lucent matrix (**A**,**D**,**E**). Darkly stained mitochondria ((**B**,**E**), red dashed circles) showed signs of mitophagy. Cell membrane (**A**,**D**) exhibited blebbing (purple arrow) and apoptotic bodies (orange arrow) were visible intercellular space. Dilated ER was shown in both treatments ((**A**–**E**), red arrow). Karyorrhexis (blue dashed circle) was observed in some cells (**F**). C, Cytoplasm. N, nucleus. Scale bar = 1.0 µm (**A**). Scale bar = 500 nm (**B**,**C**,**E**). Scale bar = 2.0 µm (**D**). Scale bar = 5.0 µm (**F**).

Ultrastructural features were observed with transmission electron microscopy. Nuclear membrane damage, apoptotic bodies, membrane blebbing, and dilated endoplasmic reticula and Golgi apparatus were visible with 25(OH)D_3_ treatment (Figure 4). In addition, mitochondria exhibited cristae disarrangement, partial and total cristolysis, and an electron lucent matrix with 25(OH)D_3_ treatments (Figure 5).

**Figure A1:**
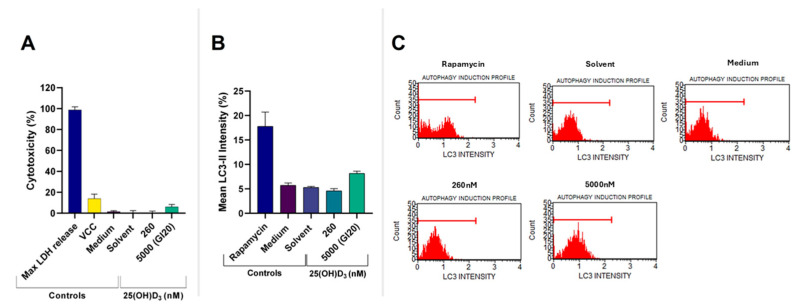
Detection of necrosis and autophagy-mediated cell death in HeLa control and experimental cultures. Analysis of necrosis (**A**) by the lactate dehydrogenase assay revealed insignificant changes in cell cytotoxicity. Detection of autophagy-mediated cell death by flow cytometry revealed insignificant LC3-II activity (**B**,**C**). Rapamycin was utilized as a positive control for autophagy-mediated cell death. Data represent mean ± SEM obtained from three independent biological replicates.

Both biochemical and morphological data did not identify autophagy-mediated cell death or necrosis in (Appendix A Figure A1).

### 2.3. 25(OH)D_3_ Regulates VDMS in HeLa Cells

**Figure 6:**
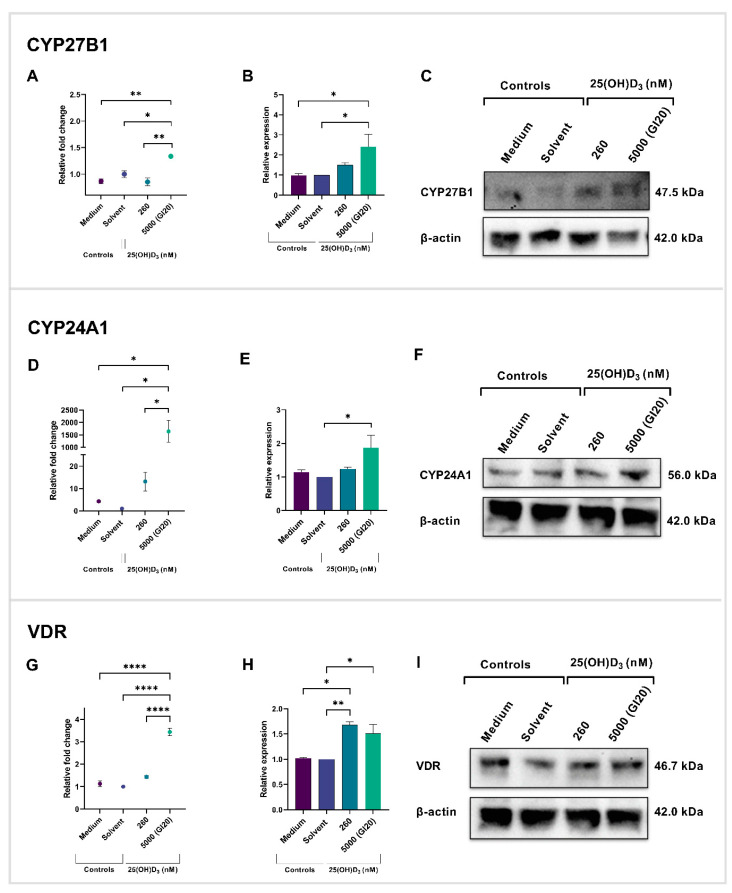
Gene and protein expression of VDMS in HeLa control and experimental cultures. CYP27B1 gene (**A**) and protein (**B**,**C**), CYP24A1 gene (**D**) and protein (**E**,**F**), and VDR gene (**G**) and protein expression (**H**,**I**) were assessed by RT-qPCR and Western blotting. Data represent mean values from three biological repeats. Full-length blots/gels are presented in Supplementary Figure S2. (* *p* < 0.05; ** *p* < 0.01; **** *p* < 0.0001).

Total RNA extracts from cell cultures were synthesized into cDNA. The cDNA was amplified with real-time PCR. The mRNA expression for CYP27B1 was significantly upregulated at a 5000 nM treatment (1.335 ± 0.01878) in comparison to the solvent control (1.000 ± 0.06477, *p* = 0.0151) and 260 nM treatment (0.8532 ± 0.07261, *p* = 0.0015) (Figure 6A). For CYP24A1, 5000 nM treatment (1641 ± 444.4) significantly upregulated gene expression relative to the solvent control (1.000 ± 0.05114, *p* = 0.0107) and 260 nM treatment (13.07 ± 4.195, *p* = 0.0205) (Figure 6D). The VDR mRNA was significantly upregulated with 5000 nM treatment (3.431 ± 0.1673) in comparison to the solvent control (1.000 ± 0.02935, *p* < 0.0001) and 260 nM treatment (1.433 ± 0.06940, *p* < 0.0001) (Figure 6G). There were no significant changes in the expression of the three VDMS genes at the 260 nM 25(OH)D_3_ treatment dose relative to control cultures.

Total protein was extracted from experimental and control cultures. Proteins were separated with gel electrophoresis and identified with immunoblotting. There was a significant increase in CY27B1 (*p* = 0.0210) (Figure 6B,C), CYP24A1 (*p* = 0.0482) (Figure 6E,F), and VDR (*p* = 0.0344) (Figure 6H,I) protein expression with 5000 nM 25(OH)D_3_ treatment. Treatment at 260 nM only increased VDR protein expression (*p* = 0.0091).

## 3. Discussion

The active vitamin D hormone, 1,25(OH)_2_D_3_, has demonstrated the ability to inhibit cell growth and proliferation, and induce cell death in various cancer cell lines. In this study, we demonstrate that its precursor, 25(OH)D_3_, decreased cell count and viability, induced apoptotic cell death, and upregulated the gene and protein expression of VDMS enzymes (CYP27B1 and CYP24A1) and receptor (VDR) in the HeLa adenocarcinoma cell line.

The decrease in the cell count and viability of 25(OH)D_3_-treated HeLa cells observed in this study was similar to the decreases observed in CaSki cervical cancer cells treated with high doses of cholecalciferol (100 ng/mL and 1000 ng/mL), the precursor of 25(OH)D_3_ [21]. Shruthi et al. demonstrated 50% growth inhibition of HeLa cells with 1250 μM cholecalciferol treatment [38]. Collectively, these studies demonstrate that high dose vitamin D metabolites negatively affect cell growth in cervical cancer cell lines.

In this study, there was an accumulation of cells in the sub-G_1_ population with 5000 nM 25(OH)D_3_ treatment observed with flow cytometry. The sub-G_1_ cell population is associated with apoptotic bodies, micronuclei, and nuclear fragments typically associated with nuclear damage and cell death [39]. This suggests that high dose treatments of 25(OH)D_3_ potentially cause DNA damage in the HeLa cell line via apoptosis. Similar findings were observed in the SiHa and CaSki cervical cancer cell lines treated with 1000 ng/mL cholecalciferol treatment. Also, treatment with cholecalciferol at 100 ng/mL increased the size of the sub-G_1_ cell population [19,20,21,22]. These findings identify that the increased sub-G_1_ cell fraction induced by cholecalciferol and 25(OH)D_3_ treatments of cervical cancer cells is dose-dependent and varies between cervical cancer cell lines.

The intrinsic pathway of apoptosis is characterized by the permeabilization of the outer mitochondrial membrane in response to stress or external stimuli. This in turn releases cytochrome C, which activates caspases. The activation of executioner caspases −3 and −7 results in the degradation of the nucleus and cytoplasm [40]. Apoptotic cells are also characterized by the externalization of phosphatidylserines to the outer layer of the plasma membrane. Flow cytometric analysis of the biochemical features of apoptosis in this study showed that treatment with 25(OH)D_3_ depolarized the mitochondrial membrane potential, increased phosphatidylserine externalization, and increased caspases −3 and −7 activation. Gross and ultrastructural analysis revealed that cells in 25(OH)D_3_ cultures had nuclear lysis (karyolysis), nuclear fragmentation (karyorrhexis), cytoplasmic condensation, cell membrane blebbing, and apoptotic bodies [41]. Several mitochondria in cells in the treatment cultures showed features of mitophagy. Mitophagy can lead to apoptosis when stress stimuli are prolonged. Collectively, the decrease in cell viability, increase in mitochondrial membrane depolarization, and the atypical mitochondria morphology infer that 25(OH)D_3_ induces intrinsic apoptosis in the HeLa cell line. Apoptotic cell death has been observed in cervical cancer cell lines with cholecalciferol [19,21,42], 25(OH)D_3_ [20], and 1,25(OH)_2_D_3_ treatments.

In addition, the ER was dilated in numerous cells, indicating potential ER stress. ER stress refers to the accumulation and aggregation of unfolded proteins in a cell. This is normally combated through the ER transmembrane receptors pancreatic ER kinase-like ER kinase (PERK), activating transcription factor 6 (ATF6), and inositol-requiring enzyme 1 (IRE1) [43,44]. Prolonged stress can trigger pro-apoptotic signaling by activating c-Jun N-terminal kinase (JNK) and inducing C/EBP homologous protein (CHOP). The CHOP blocks the expression of BCL2 proteins, while JNK phosphorylates Bim, leading to caspase activation [44]. Few studies have explored the relationship between vitamin D metabolites and ER stress in cancer [45,46]. Given that BCL2 proteins were not analyzed, it cannot be concluded that 25(OH)D_3_ induced ER stress and if the ER stress in turn induced apoptosis. However, the presence of ER stress could explain the differences in the relative fold changes in the VDMS genes and proteins.

The expression of VDMS can be observed in both healthy and tumorigenic cervical tissue [18]. In this study, it was found that HeLa cells display an autocrine response to 25(OH)D_3_ treatment. Treatment with 5000 nM of 25(OH)D_3_ significantly increased the gene and protein expression of CYP27B1, CYP24A1, and VDR in HeLa cells. In contrast, treatment at the physiological dose level (260 nM) resulted in a significant increase in VDR protein expression without affecting the mRNA transcript level, and it did not alter the gene and protein expression of CYP27B1 and CYP24A1. This suggests that treatment did not influence the activation and catabolism of 25(OH)D_3_ at the physiological dose.

The upregulation of the CYP27B1 gene and protein expression was observed with 5000 nM treatment, and suggests the autocrine conversion of 25(OH)D_3_ to 1,25(OH)_2_D_3_. Comparable results were observed in SiHa cervical cells treated with 2500 nM 25(OH)D_3_, which induced a five-fold increase in CYP27B1 gene expression [20]. These findings suggest that supraphysiological doses enhance CYP27B1 expression in cervical cell lines. These results, in combination with cell count, viability, and cell death data, suggest that high dose treatments lead to a negative feedback regulation of 25(OH)D_3_ levels. However, there is a possibility that treatment with a supraphysiological dose may result in the direct inhibition of cell growth and induction of apoptosis by inactivated 25(OH)D_3_, without activation through 1αOHase (CYP27B1). Further investigations are required to conclude this mechanism of action. Additionally, this study revealed that treating HeLa cells with a physiological dose did not have a statistically significant effect on CYP27B1 mRNA and protein expression. Similarly, Kloss et al. reported that 100 nM of 25(OH)D_3_-treated HeLa cells did not display altered CYP27B1 gene expression [47]. Supraphysiological levels of 25(OH)D₃ have been shown to inhibit cell proliferation and promote cell death independently of its activation by CYP27B1. In LNCaP prostate adenocarcinoma cells, treatment with 100 nM 25(OH)D₃, alongside the CYP27B1 inhibitor genistein, resulted in a marked reduction in cell growth, suggesting that 25(OH)D₃ can exert direct anti-proliferative effects [13]. These findings support the potential use of 25(OH)D_3_ precursors as therapeutic agents in cervical cancer, acting either directly or through autocrine activation of CYP27B1. 

CYP24A1 catabolizes 25(OH)D_3_ and 1,25(OH)_2_D_3_. Our results suggest that a supraphysiological dose induces catabolism of 25(OH)D_3_ by CYP24A1. Numerous studies have displayed that CYP24A1 expression is induced by 1,25(OH)_2_D_3_ [48]. Interestingly, studies have revealed that inhibiting or knocking down CYP24A1 significantly impedes tumor growth, whereas overexpressing CYP24A1 promotes tumor progression and metastasis [49,50,51]. In this study, treatment with 5000 nM resulted in the overexpression of the CYP24A1 gene and significantly increased protein expression. Although cell growth was inhibited, the maximum inhibition observed was approximately 20%, suggesting that CYP24A1 may exert oncogenic effects on the HeLa cells with high doses of 25(OH)D_3_. The CYP24A1 is silenced in some cancer cells, such as tumor-derived endothelial cells of the colon, resulting in tumor-suppressive effects with vitamin D treatment [52].

The expression of target genes of vitamin D hormones is primarily regulated by VDR. Both 25(OH)D_3_ and active hormone 1,25(OH)_2_D_3_ can bind to VDR; however, they exhibit different potencies [53]. We noted the increase in VDR protein expression with physiological and supraphysiological doses of 25(OH)D_3_, indicating that VDR mediates cell growth inhibition and cell death mechanisms. Interestingly, a physiological dose (260 nM) of 25(OH)D_3_ did not significantly change VDR expression in SiHa cervical cells, and a supraphysiological treatment dose significantly downregulated VDR gene and protein expression (*p* = 0.0380) [20], suggesting that intracellular signaling by VDR is cell line- and dose-dependent.

In summary, our study demonstrates that 25(OH)D_3_ exerts a pro-apoptotic effect on the cervical adenocarcinoma cell line, HeLa, evidenced by the upregulation of biochemical and morphological markers of apoptotic cell death. These effects are mediated by physiological and supraphysiological treatment doses at 260 nM and 5000 nM of 25(OH)D_3_. The autocrine metabolism of 25(OH)D_3_ is regulated in experimental cultures by 25(OH)D_3_ treatment doses, which upregulate activation and signaling via increased CYP27B1 and VDR expression, respectively. Notably, the catabolic enzyme, CYP24A1, is also upregulated at high dose treatments, which most likely attenuates the anti-cancer action of 25(OH)D_3._

Further investigations are required to determine whether the actions of 25(OH)D_3_ on HeLa cells are mediated solely by VDMS or in combination with other anti-cancer molecular mechanisms exerted by 25(OH)D_3_. Additionally, clinical observational studies exploring the role of vitamin D levels in human patients with cervical cancer are warranted.

## 4. Materials and Methods

### 4.1. Cell Culture and Treatments

The HeLa cell line (CCL-2™) was obtained from the American Type Culture Collection (ATCC, Manassas, VA, USA) and grown in Dulbecco’s Modified Eagle Medium (DMEM) supplemented with 10% foetal bovine serum (ThermoFisher Scientific, Waltham, MA, USA), 100 µg/mL streptomycin, 100 U/mL penicillin G, 2.5 mM L-glutamine, and 250 µg/L fungizone (Sigma-Aldrich, St. Louis, MO, USA). Cell cultures were incubated in a humidified incubator at 5% CO_2_ and 37 °C.

The cells were treated with 25(OH)D at 260 nM and 5000 nM for 72 h. The solvent control culture was treated with diluent ethanol diluted in culture medium to a final concentration of 0.5% (*v*/*v*) ethanol. This corresponds to the ethanol in the 5000 nM 25(OH)D_3_ treatment dose. The medium control contained HeLa cells with medium only. Each assay was conducted in triplicate on three independent experiments.

### 4.2. Crystal Violet Assay

HeLa cells (25,000 cells/mL) were washed with 1x phosphate buffered saline (PBS), pH 7.4, and were stained using a published method [54]. The optical density was measured at 570 nm on the ELx800 Universal Microplate Reader (Bio-Tek Instruments Inc., Winooski, VT, USA).

### 4.3. AlamarBlue Assay

HeLa cells (25 000 cells/mL) were incubated with alamarBlue Cell Viability Reagent (Thermo Fisher Scientific, Waltham, MA, USA) diluted in culture medium in a 1:10 ratio for 4 h at 5% CO_2_ and 37 °C, protected from light. Absorbance was measured at 570 nm and 600 nm using the ELx800 Universal Microplate Reader (Bio-Tek Instruments Inc., Winooski, VT, USA).

### 4.4. Cell Cycle Distribution by Flow Cytometric Analysis

A cell count of 1 × 10^6^ cells was harvested using trypsin and resuspended in culture medium. The cells were centrifuged at 300× *g* for 5 min and the pellets were re-suspended in 1X PBS (pH 7.4). The cells were fixed in ice-cold 70% (*v*/*v*) ethanol and frozen at −20 °C for 3 h. The fixed cells (200 µL) were washed and resuspended in 1X PBS (pH 7.4) and stained with Muse™ Cell Cycle Reagent at room temperature for 30 min and protected from light. The samples were then analyzed in a Guava^®^ Muse™ Cell Analyzer (Luminex, Austin, TX, USA) using Muse™ software v1.8.0.3 (Windows, Luminex Corporation, Austin, TX, USA). The sub-G_1_ was calculated as follows: 100 − (G_0_/G_1_ population + S population + G_2_/M population).

### 4.5. Mitochondrial Membrane Disruption Analysis with Flow Cytometry

A cell count of 2 × 10^6^ was harvested and resuspended in 1X Assay Buffer [53]. The positive control was treated with actinomycin D at a final concentration of 1 µg/mL. Thereafter, the cell suspension was incubated with 95 μL of the Mito Potential working solution (Luminex) for 20 min in a 37 °C incubator with 5% CO_2_. Then, 5 μL 7-aminoactinomycin D (7-AAD) reagent was added to each sample and incubated for 5 min at room temperature. Samples were analyzed in a Guava^®^ Muse™ Cell Analyzer (Luminex, Austin, TX, USA) using Muse™ v1.8.0.3. software (Windows, Luminex Corporation, Austin, TX, USA) [55].

### 4.6. Annexin V Staining by Flow Cytometry

A cell count of 2 × 10^6^ cells was harvested and resuspended in DMEM. Actinomycin D (1 µg/mL) was used to treat the positive control. Then, 100 μL cell suspension was mixed with 150 μL of the Annexin V & Dead Cell Reagent (Luminex, Austin, TX, USA) and incubated at room temperature for 20 min, protected from light. Samples were analyzed in a Guava^®^ Muse™ Cell Analyzer using Muse™ software v1.8.0.3. (Windows, Luminex Corporation, Austin, TX, USA) [55]

### 4.7. Caspase-3/7 Assay with Flow Cytometry

A cell count of 2 × 10^6^ cells was harvested and resuspended in 1X Assay Buffer. Actinomycin D (1 µg/mL) was used to treat the positive control. Thereafter, 50 μL cell suspension was mixed with 5 μL of Caspase-3/7 Reagent working solution (Luminex, Austin, TX, USA) in microcentrifuge tubes. The caps of the tubes were loosened, and the tubes were incubated for 30 min at 37 °C and 5% CO_2_ in a humidified incubator. After incubation, 150 μL of Caspase 7-AAD (Luminex) working solution was added to each sample and mixed before incubating at room temperature for 5 min, protected from light. The samples were analyzed in a Guava^®^ Muse™ Cell Analyzer using Muse™ software v1.8.0.3. (Windows, Luminex Corporation, Austin, TX, USA) [55].

### 4.8. Autophagy LC3-II Assay with Flow Cytometry

A cell count of 5000 cells/mL cells was washed with 1X PBS. Rapamycin (200 nM) was used to treat the positive control to a final concentration. Then, a solution of 200 μL of DMEM and 0.2 μL of Autophagy Reagent A (1:1000 dilution) (Luminex, Austin, TX, USA) was added to each sample. The samples were incubated at 37 °C for 6 h to induce autophagy. After incubation, the cells were washed with 1X PBS, harvested using trypsin, and resuspended in 1 × PBS in Eppendorf tubes. The sample tubes were centrifuged at 300× *g* for 5 min at 4 °C. The supernatant was discarded and 5 μL of Anti-LC3 Alexa Fluor™ 555 (Luminex) and 95 μL of 1X Autophagy Reagent B (Luminex) were added to each sample tube. The samples were incubated on ice for 30 min, protected from light. Thereafter, the samples were centrifuged at 300× *g* for 5 min at 4 °C and the supernatant was discarded. The samples were resuspended in 200 μL 1X Assay Buffer and analyzed immediately in a Guava^®^ Muse™ Cell Analyzer using Muse™ v1.8.0.3. software (Windows, Luminex Corporation, Austin, TX, USA).

### 4.9. Lactate Dehydrogenase (LDH) Assay

Cells were seeded at 5000 cells/well and 50 μL of culture medium was transferred to a clean 96-well culture plate, followed by 72 h incubation with treatment. To each sample, 50 μL of LDH buffer was added. The samples were gently mixed for 30 s and then incubated at room temperature for 30 min, protected from light. Thereafter, 50 μL of 1 M acetic acid was added to each well. The absorbance was measured at 490 nm using the ELx800 Universal Microplate Reader (Bio-Tek Instruments Inc., Winooski, VT, USA).

### 4.10. Brightfield Microscopy

A total cell count of 5 × 10^4^ cells/coverslip was seeded on sterile glass coverslips and incubated for 72 h with treatments or control cultures. The cells were gently rinsed with 1X PBS and then fixed with Bouin’s fixative (Sigma-Aldrich, St. Louis, MO, USA) for 30 min. The cells were then incubated in 70% ethanol for 20 min. Thereafter, the cells were rinsed with tap water and then stained in Mayer’s Hemalum (Sigma-Aldrich) for 20 min. The coverslips were rinsed using running tap water for 2 min, followed by 70% ethanol. The cells were then stained with 1% eosin (Sigma-Aldrich) for 7 min. Thereafter, the cells were rinsed with 70% ethanol, 95% ethanol, 100% ethanol, and xylol (Sigma-Aldrich), successively for 5 min each. This process was repeated once. The coverslips were mounted to microscope slides with Entellan mounting fluid (Sigma-Aldrich) and left to dry. The samples were viewed, and images were captured using an Olympus DP74 camera. Cells were counted, measured, and analyzed for gross morphological changes using ImageJ 1.53e (Java 1.8.0_172; National Institutes of Health, Bethesda, MD, USA).

### 4.11. Transmission Electron Microscopy (TEM)

HeLa cells were prepared following the steps adopted from a published protocol [56]. The cells were washed with 1X PBS and collected in Eppendorf tubes. The cells were centrifuged for 3 min at 300× *g*, and then the supernatant was discarded. The cells were then fixed in Palade’s fixative for 1 h at room temperature. Thereafter, the fixative was removed, and the cells were incubated in 50% alcohol for 15 min. This step was repeated using 70% alcohol for 15 min, 96% alcohol for 30 min, twice with 100% alcohol for 30 min, and then propylene oxide for 30 min. Then, cells were incubated in a 1:1 mixture of propylene oxide and embedding medium for 90 min. Thereafter, the HeLa cells were centrifuged for 3 min and transferred to gelatine capsules. Embedding media was added to the capsules, and they were placed in a 65 °C oven overnight to allow the media to polymerise.

A Reichert-Jung Ultracut ultramicrotome was used for sectioning and samples were collected on Agar Grids 300 Mesh Copper disks (Wirsam Scientific and Precision Equipment (Pty) Ltd., Johannesburg, South Africa). The samples were stained with UA-Zero^®^ EM Stain (Wirsam Scientific) for 1 h, followed by lead citrate (Wirsam Scientific) for 3 min. The samples were viewed using a camera from JEOL Ltd. (Tokyo, Japan) and analyzed for ultrastructural changes.

### 4.12. RNA Isolation

Total RNA was extracted from HeLa cells seeded at 2 × 10^5^ cells/mL. The cells were washed with cold 1X PBS and lysed with 500 μL lysis reagent (Qiagen, Hilden, Germany). The samples were incubated on ice for 5 min. Cell residue was collected in Eppendorf tubes and frozen for 1 h at −80 °C. After thawing, 100 μL chloroform was added, and the samples were vigorously mixed and centrifuged at 12,000× *g* for 15 min at 4 °C. The upper phase was collected and incubated for 1 h at −80 °C with isopropanol. The samples were then centrifuged at 12,000× *g* for 20 min at 4 °C, and the supernatant was discarded. The pellet was gently washed with 75% cold ethanol and then centrifuged at 7400× *g* for 15 min at 4 °C. The ethanol was discarded, and the pellet was allowed to air dry for 5 min before resuspending in 15 μL of nuclease-free water (Celtic Molecular Diagnostics, Cape Town, South Africa). The samples were incubated at room temperature for 3 min and then placed on ice. The concentration and integrity of the isolated RNA was analyzed using an Optizen NanoQ™ (KLAB Keen Innovative Solutions, Daejeon, Republic of Korea). The total RNA was stored at −80 °C.

### 4.13. RT-qPCR

Reverse transcription was performed according to the iScript™ Reverse Transcription Supermix protocol (Bio-Rad, Johannesburg, South Africa) using 4000 ng RNA in a Rotor-Gene Q thermal cycler (QIAGEN, Hilden, Germany). The synthesized cDNA was then diluted with 40 μL of nuclease-free water, aliquoted in 20 μL volumes, and stored at −80 °C. The cDNA was amplified according to the iTaq™ Universal SYBR^®^ Green Supermix kit manufacturer instructions (BioRad). The reaction procedure was as follows: 1 cycle of 95 °C for 30 s, 40 cycles of 95 °C for 15 s, 64 °C for 15 s, and 72 °C for 10 s. All primer sequences were obtained from previously published work and were purchased from Inqaba Biotechnology (Pretoria, South Africa) [57,58]. Melt curve analysis was as follows: 65–95 °C in 0.5 °C increments at 5 s per step. The fold change in gene expression was determined using the delta-delta Ct method.

### 4.14. Western Blot

Total protein was isolated from HeLa cells using the M-PER™ Mammalian Protein Extraction Reagent (ThermoFisher, Waltham, MA, USA) and 100x EDTA-free Halt™ Protease Inhibitor Cocktail (ThermoFisher) in a 100:1 ratio. The protein lysates were quantified using the Pierce™ bicinchoninic acid (BCA) protein assay kit (ThermoFisher), as described by the manufacturer. The protein concentrations were standardized to 20 μg using M-PER Mammalian Protein Extraction Buffer. To each sample, 6.3 μL of 4X NuPAGE™ LDS Buffer (ThermoFisher) and 0.626 μL of 2-Mercaptoethanol (Sigma-Aldrich) were added. The samples were then denatured at 95 °C for 7 min. SDS-page was performed at 200 volts for 80 min. A wet sandwich transfer onto a PVDF membrane was conducted for 90 min at 110 volts. The membrane was then blocked with 2.5% BSA in 0.2% PBS-Tween for 1 h. The membrane was probed with the following primary antibodies (anti-rabbit) overnight at 4 °C: CYP27B1, CYP24A1, and VDR. The secondary antibody consisted of the HRP-conjugated goat anti-rabbit antibody. HRP-conjugated anti-β-actin was used as a control.

### 4.15. Statistics

We evaluated the differences between experimental and control cultures by one-way ANOVA followed by a post hoc Bonferroni test, and *p* ≤ 0.05 was considered significant. All data are expressed as the mean ± SEM. All computations were performed using GraphPad Prism v9.4.1 (GraphPad Software Inc., San Diego, CA, USA).

## 5. Conclusions

25(OH)D_3_ inhibits cell growth and induces apoptotic cell death in the HeLa cell line via VDMS in an autocrine manner. Cell growth and survival is regulated with 25(OH)D_3_ treatment, through activation by CYP27B1 and VDR. CYP24A1 catabolizes 25(OH)D_3_ and potentially exerts oncogenic effects with high dose treatments. Currently, cervical cancer is treated with surgery and chemo-radiotherapy. 25(OH)D_3_ holds immense potential as a complementary therapeutic treatment for cervical cancer owing to its low calcemic effect. Further investigations are essential to explore the effects in vivo and conduct translational observational studies in patient populations.

## Data Availability

All data included in this study are available upon request by contact with the corresponding author.

## Supplementary Materials

The following supporting information can be downloaded at: https://www.mdpi.com/article/10.3390/ijms26094008/s1.

## Abbreviations

The following abbreviations are used in this manuscript:

1,25(OH)_2_D_3_	1,25-dihydroxycholecalciferol
25(OH)D_3_	25-hydroxycholecalciferol
ANOVA	Analysis of variance
BSA	Bovine serum albumin
CYP24A1	Cytochrome P450 25-hydroxyvitamin D-24-hydroxylase
CYP27B1	Cytochrome P450 25-hydroxyvitamin D-1α-hydroxylase
ER	Endoplasmic reticulum
HRP	Horseradish peroxidase
PBS	Phosphate buffered saline
PVDF	Polyvinylidene fluoride
SEM	Standard error of mean
VDMS	Vitamin D metabolizing system
VDR	Vitamin D receptor
