# Supplementary material for: Induction of Cell Death and Regulation of Autocrine Vitamin D Metabolism in Cervical Cancer by Physiological and GI20 Doses of 25-Hydroxycholecalciferol
Source: Int J Mol Sci. 2025 Apr 24;26(9):4008. doi: 10.3390/ijms26094008 (PMC12071354; doi:10.3390/ijms26094008)
Supplement: Supplementary file 1 [file ijms-26-04008-s001.zip › ijms-3541676-supplementary.pdf]

Supplementary Data

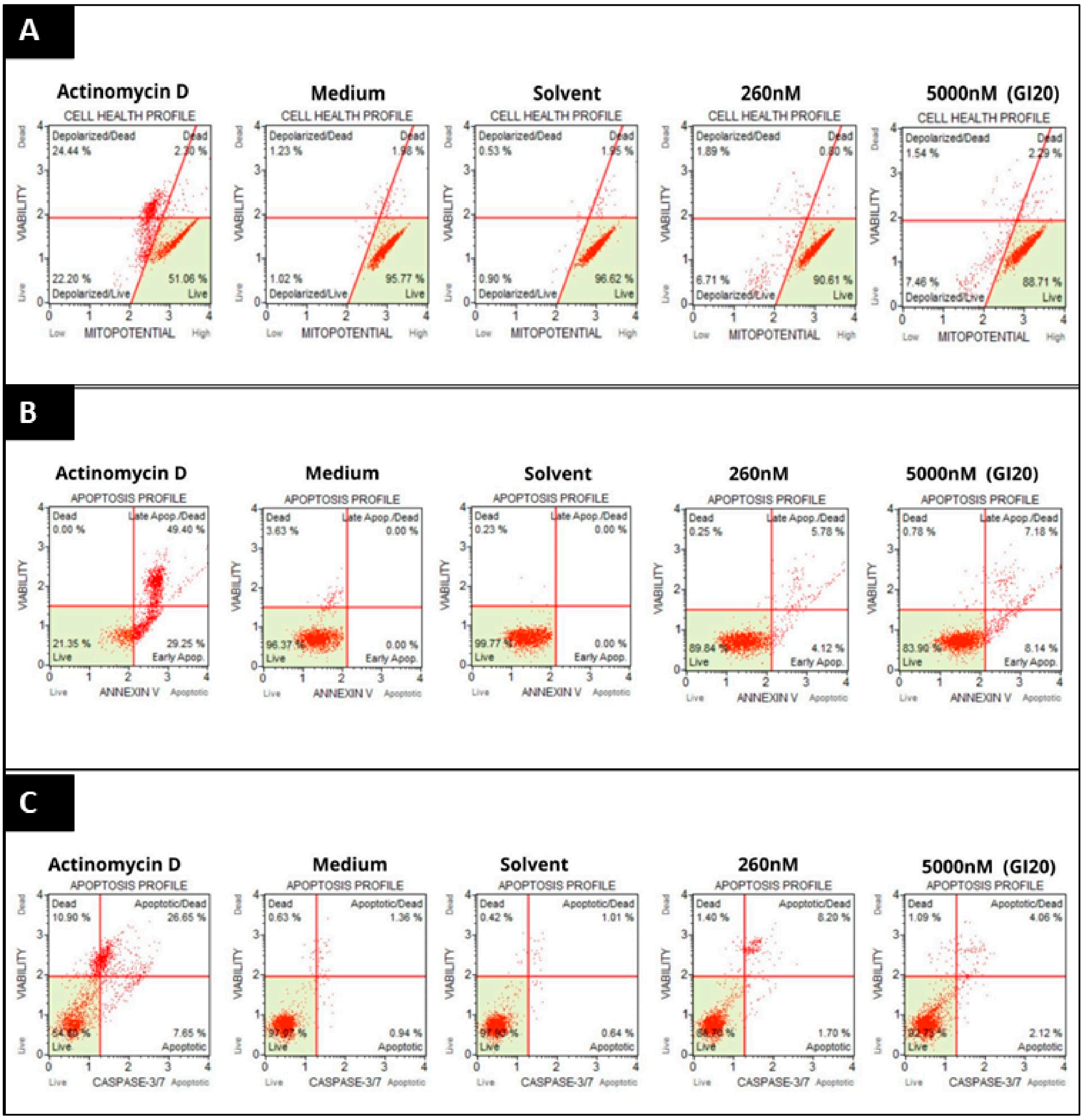

Figure S1. Representative dot-plot diagrams for detection of biochemical apoptosis with three flow cytometric assays. Changes in (A) mitochondrial membrane potential, (B) phosphatidylserine externalisation and (C) caspase -3 and -7 activation in experimental and control HeLa cultures were assessed by flow cytometry. Actinomycin is the positive control for apoptosis.

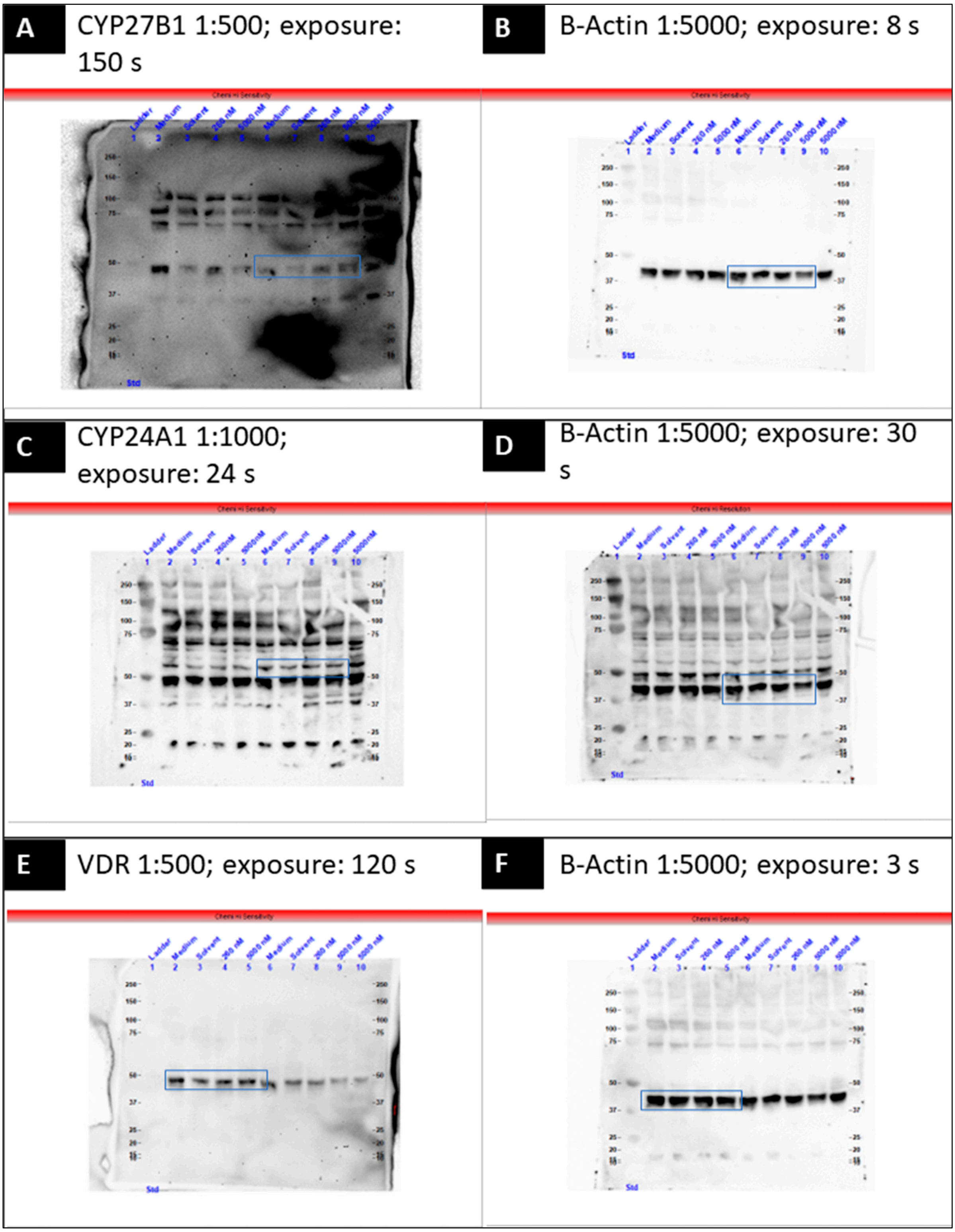

Figure S2. Representative Western blots for VDMS proteins. Western blot analysis for the VDMS proteins included: (A) CYP27B1, (C) CYP24A1, (E) VDR and the associated housekeeping protein β-Actin (B, D and F respectively) for each of the VDMS proteins. The representative proteins are indicated in blue squares and the the lanes labelled for control and experimental cultures. The protein molecular weight ladder is indicated for each blot.
